# First Gynogenesis of *Vanilla planifolia* for Haploid Production and Ploidy Verification Protocol

**DOI:** 10.3390/plants13131733

**Published:** 2024-06-23

**Authors:** Manuel Gastelbondo, Ursula Nicholls, Sisi Chen, Alan Chambers, Xingbo Wu

**Affiliations:** 1 Plant Breeding Graduate Program, Tropical Research and Education Center, University of Florida, 18905 S.W. 280 Street, Homestead, FL 33031, USA; gastelbondom@ufl.edu (M.G.); sisi.chen@ufl.edu (S.C.); 2 Horticulture Department, Tropical Research and Education Center, University of Florida, 18905 S.W. 280 Street, Homestead, FL 33031, USA; unicholls@unal.edu.co (U.N.); alan.chambers@keygene.com (A.C.); 3 Environmental Horticulture Department, Tropical Research and Education Center, University of Florida, 18905 S.W. 280 Street, Homestead, FL 33031, USA

**Keywords:** *Vanilla planifolia*, haploids, gynogenesis, tissue culture, ploidy

## Abstract

Vanilla orchids are members of the Vanilloideae orchid subfamily, and they hold significant economic value as a spice crop in tropical regions. Despite the presence of 180 known species within this subfamily, commercial production focuses on only three species (*Vanilla planifolia*, *V. odorata*, and *V. pompona*) and one hybrid (*V. × tahitensis*), prized for their aromatic qualities and bioactive compounds. Limited modern breeding initiatives have been undertaken with vanilla orchids, although recent advancements in genomic research are shedding light on this crop’s potential. The protracted breeding cycle of vanilla, coupled with increasing demand for germplasm, underscores the importance of research and breeding efforts in vanilla. This paper outlines a protocol for haploid production in *V. planifolia* using unfertilized ovaries in tissue culture conditions. Additionally, we present a methodology to confirm the haploid nature of putative haploid lines through stomatal size comparison, chromosome counting, and flow cytometry analysis, proving the successful development of haploid vanilla plants. These findings contribute to the advancement of breeding programs and genetic improvement strategies for the vanilla industry.

## 1. Introduction

Natural vanilla is highly valued as a culinary delicacy and commonly available in the form of cured vanilla capsules or their extracts [1]. However, the conventional propagation methods for vanilla, particularly vegetative propagation through cuttings, pose challenges such as limited genetic diversity, labor intensity, and resource requirements [2]. Additionally, existing commercial vanilla lines represent only a fraction of the genetic potential found in wild populations, requiring modern breeding approaches to fully achieve the crop’s potential [3]. For example, the use of clonal material from a narrow genetic background in vanilla has resulted in high susceptibility to *Fusarium* root and stem rot, which has reduced the world vanilla production by half, even though wild *Vanilla pompona* and hybrids have proven to be resistant to the disease [4].

Tissue culture has improved the propagation efficiency of many plants, including vanilla [5]. Even so, the bottleneck remains at the initiation phase. In vanilla orchids, explants are restricted to growing tips or nodal segments for initiation. Such material takes as long as a year to establish and start a bi-monthly propagation factor of two (meaning that in two years you will get from 1 explant to 64). Also, the high rates of endophytes pose a major limitation during initiation, due to contamination. Asymbiotic seed germination offers a promising alternative, resulting in thousands or more plantlets in one year, albeit with high heterozygosity and likely trait segregation, making them unsuitable for commercial use.

Double haploid is a technique that has been successfully employed in various crops, including maize, Gentiana, flax, brassica, gerbera, chrysanthemum, and others, to obtain completely homozygous plants in one generation [6]. This technique offers a solution to the initiation bottleneck in vanilla by producing homozygous parental lines that can be used to produce homogeneous stable sexual seeds, either for hybrid production or self-pollination [7]. Additionally, cycling plants through a haploid phase can eliminate deleterious alleles that are hidden in heterozygous diploids. The reduction in deleterious alleles can increase overall plant vigor. However, there have been very few reports on haploid development in orchids [8,9,10], and none for any vanilla species to our knowledge, highlighting the need for research in this area. Double haploids also provides other advantages as a breeding tool, such as recessive trait discovery and selection, haplotype isolation, and haploid compatibility evaluation [6].

In this study, we report the successful development of haploid lines in *Vanilla planifolia* through gynogenesis for the first time, and we provide a comprehensive ploidy verification protocol. The resulting haploid plants are the first steps to produce double-haploid lines that can serve as parental lines for breeding programs that could potentially facilitate stable vanilla seed production for commercial use.

## 2. Results

From 32 ovary sections planted in vitro, three *V. planifolia* lines were recovered (Table 1). Two recovered lines were confirmed to be haploids, and the remaining one was confirmed to be a mixoploid [11]. The remaining explants continued to develop for three months but stopped growing and slowly turned brown until the tissue was completely necrotic. The surviving explants developed into protocorm-like bodies (PLBs) and eventually differentiated towards shoot clusters that could be divided and provided a source of micro-cuttings for further multiplication. Stock clonal populations were multiplied and kept for further research and breeding efforts. 

**Figure 1:**
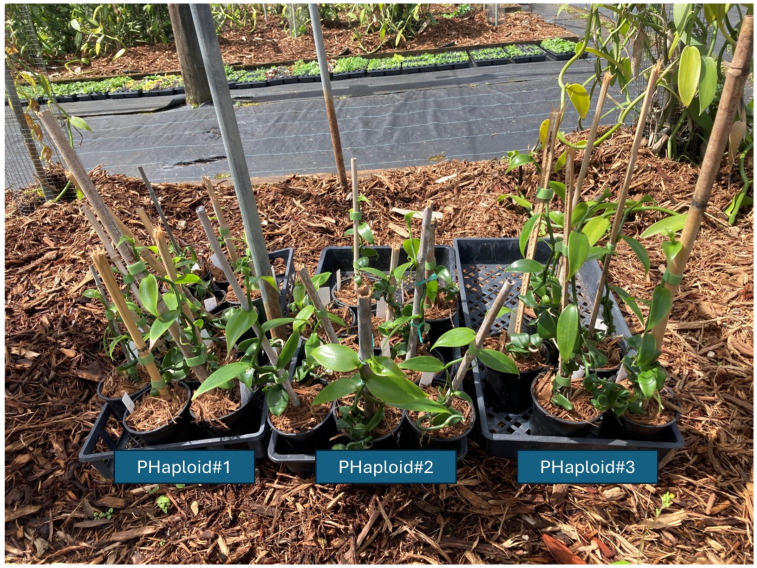
Hardened putative haploid lines adapted to shade house conditions.

During the adaptation phase, two plantlets from PHaploid#2 died; all remaining plantlets survived. The hardened plants were transplanted to individual pots with wood chip substrate and transferred to the shade house for further acclimation (Figure 1).

**Figure 2:**
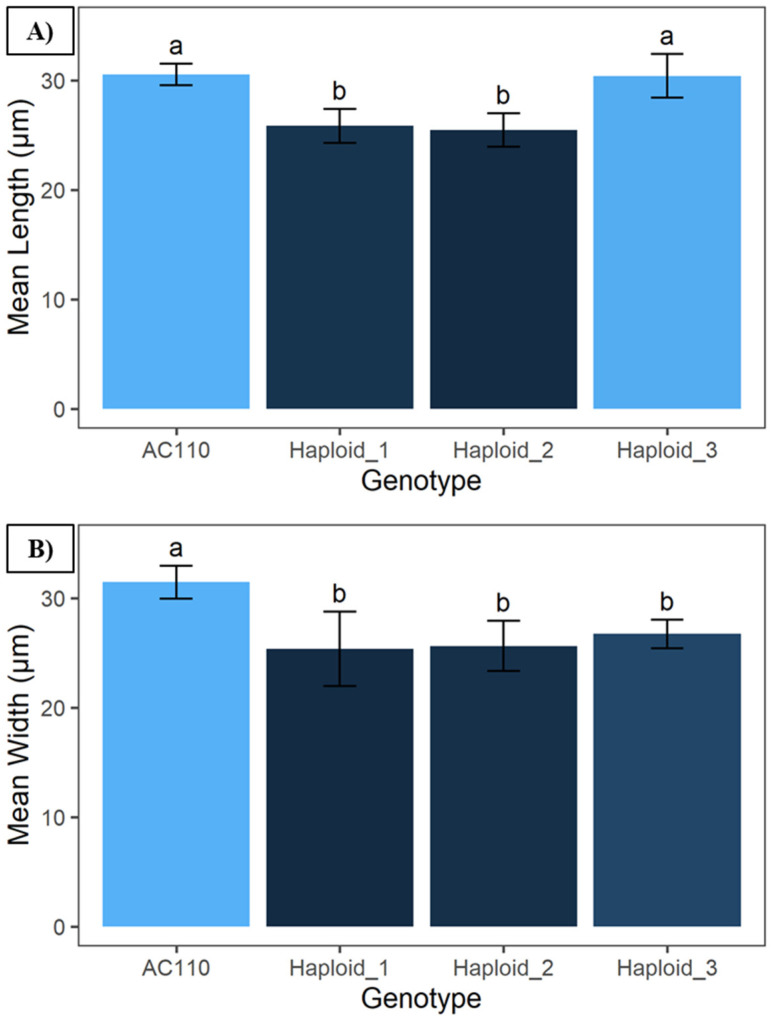
Stomatal morphology size comparison showing significant statistical differences in (**A**) stomatal length and (**B**) stomatal width for the putative haploid lines evaluated and the parental control line (AC110); the letter labels on top of the bars refer to the groups generated using Tukey’s HSD test with an alpha value of 0.01.

Stomatal morphology comparison revealed significant differences between the haploid lines and the diploid parent plant, with an alpha value of 0.01 (Figure 2). Stomatal length was significantly different for the verified haploids in comparison to the diploid parent plant (AC110) and the mixoploid line (PHaploid#3). On the other hand, all three putative haploids were significantly different with regards to stomatal width in comparison to the diploid parent plant. 

**Figure 3:**
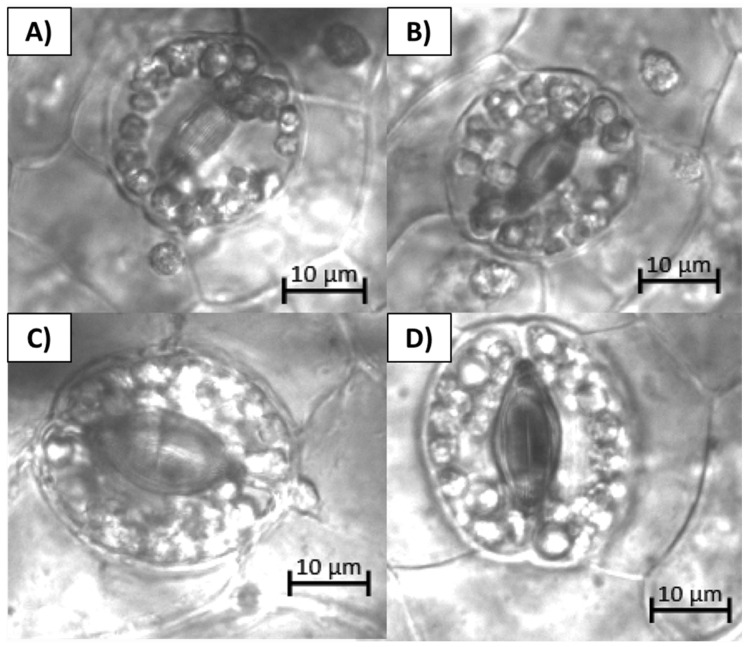
Stomatal morphology comparison of putative haploid lines and the parent plant with plastids in stomatal guard cells: (**A**) Stomata from PHaploid#1. (**B**) Stomata from PHaploid#2. (**C**) Stomata from PHaploid#3. (**D**) Stomata from the parent plant (AC110).

Stomatal size and the number of chloroplasts in the guard cells were found to be positively correlated with the ploidy level and have been used as a routine technique to identify haploid lines in different crops [12,13,14,15,16,17]. There was a qualitative appreciation of the reduced number of chloroplasts in the guard cells of the putative haploids in comparison to the control parent plant and the mixoploid samples (Figure 3).

Chromosome counting supported the previously mentioned differences, indicating different chromosome numbers for two of the putative haploid lines compared to the parent plant and the mixoploid line. The parent plant (AC110) was found to have a variable somatic number of chromosomes, with 2n counts of 28 and 32 for different somatic cells and a constant chromosome count of n = 16 in its pollen cells; this was also the case for PHaploid#3. The other *V. planifolia* accession (Daphna) had a constant chromosome number of 28 in all of the somatic cells and n = 14 chromosomes in all of the pollen cells counted (Table 2). 

**Figure 4:**
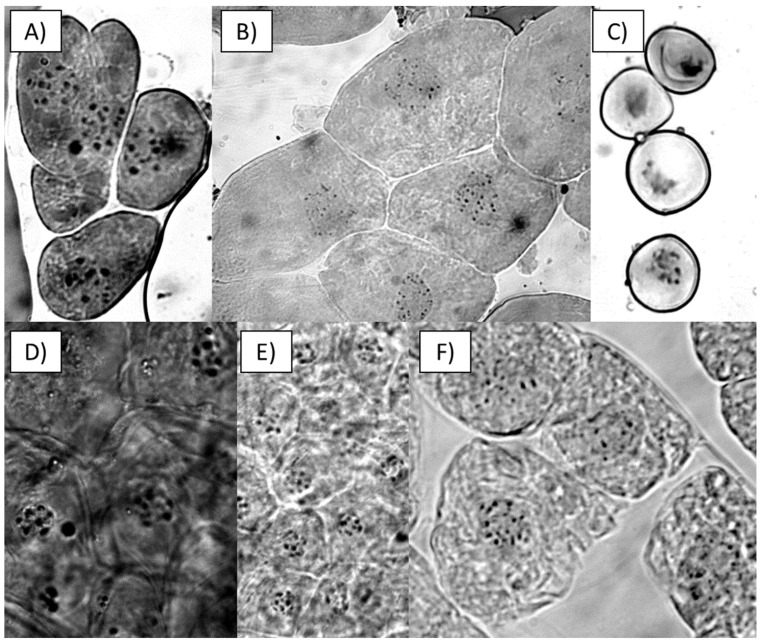
Chromosome images: (**A**) Parent plant (AC110) root tip. (**B**) Parent plant (AC110) apical meristem. (**C**) Parent plant (AC110) pollen. (**D**) PHaploid#1 root tip. (**E**) PHaploid#2 root tip. (**F**) PHaploid#3 root tip.

Pollen cells from the parent plant and the other *V. planifolia* accession (Daphna) were used as controls for comparison with the root tip cells from the putative haploid lines (Figure 4). The chromosome counts for both accessions were congruent with other cytogenetic studies in *V. planifolia* that state the occurrence of variable chromosome counts in mixoploid vanilla individuals [18]. 

The use of tissue-cultured plants made it possible to select fresh, actively growing root tips for the chromosome counting. Available in vitro plants for the putative haploid lines, as well as for the parent plant, were initiated and kept under tissue culture. Having the plant material in tissue culture was fundamental for the required tissue type for chromosome counting, as well as for flow cytometry genome size estimation. 

Flow cytometry analysis further corroborated the identification of the haploid lines. The diploid *V. planifolia* samples consistently provided estimated 2C values that ranged from 4.3 to 4.5 Gb, which is consistent with other genome size estimations of *V. planifolia* [18,19], whilst two of the putative haploid lines showed half of the normal diploid size (between 1.68 Gb and 2.05 Gb). The genome size of PHaploid#3 was broader but close to the diploid range (between 1.96 Gb and 4.81 Gb) (Table 3).

**Figure 5:**
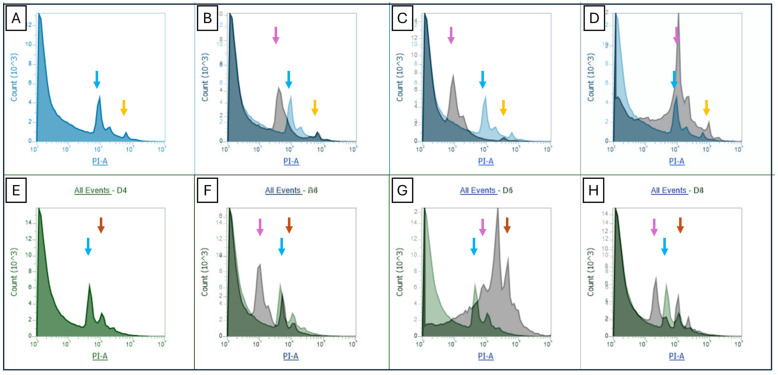
Flow cytometry histograms: (**A**) Parent plant (AC110) (blue arrow) with wheat as an internal standard (yellow arrow). (**B**) PHaploid#1 (pink arrow) superposed to the parent plant histogram. (**C**) PHaploid#2 (pink arrow) superposed to the parent plant histogram. (**D**) PHaploid#3 (pink arrow) superposed to the parent plant histogram. (**E**) Parent plant (AC110) (blue arrow) with Barley “Butta 12” as an internal standard (red arrow). (**F**) PHaploid#1 (pink arrow) superposed to the parent plant histogram. (**G**) PHaploid#2 (pink arrow) superposed to the parent plant histogram. (**H**) PHaploid#3 (pink arrow) superposed to the parent plant histogram.

The flow cytometry histograms provide strong evidence of the ploidy differences amongst the samples. The use of internal standards allowed us to compare multiple runs and overlap control runs with sample runs. As the estimations with the three internal standards were consistent in the genome sizes, the haploid nature of two of the putative haploid lines was further upheld (Figure 5). 

## 3. Discussion

The shift from clonal propagation towards homogeneous sexual seed propagation in vanilla orchids is expected to increase the availability of new genotypes for evaluation or commercial use. Haploids might also be of value for precise haplotype sequencing endeavors. The segregation patterns of haplotypes and crossing over events can be addressed for specific genes, for example, disease resistance genes or other traits of importance. 

With relation to the tissue culture protocol, it is important to mention that only ovaries from immature floral buds (stages 5 and 6) resulted in ovule development. All other explants failed to develop or grow in vitro. Our results suggest that small, undeveloped floral buds are better explants than fully developed, unpollinated flowers for haploid generation. Explant tissue was highly susceptible to oxidation during the first month of development, and constant transfer was required to avoid necrosis. The susceptibility of this crop to oxidation during initiation is a limiting factor affecting the efficiency of haploid induction using the approach mentioned here. However, it is worth mentioning that the use of antioxidants or other additives to increase line recovery success was not evaluated in this study, which could be further studied to increase the success rate. Individualized ovules did not develop unless kept attached to the ovary until they swelled and started to resemble PLBs. No explants developed if kept under light; a stratification of complete darkness for the first eight months was required.

To evaluate the ploidy of the recovered lines, the first step was morphological comparisons. This is a practical approach with low cost that can be employed as a first screening for further confirmation. Stomatal morphology is correlated with the ploidy level and has been successfully used in many crops to identify haploid lines [14,20]. Our study further supports this correlation between stomatal morphology and ploidy level, opening the possibility for its use in vanilla for haploid line validation. Stomatal guard cell chloroplast counting has also been employed to identify ploidy changes in plants, due to a positive correlation between the number of chloroplasts in the guard cells and the ploidy level [12,16]. The chloroplasts were difficult to quantify due to their abundance and overlapping with each other. It is known that orchid chloroplasts can move from cell to cell as a response to light stimulus [17]. These unique biological features are also present in vanilla and pose a challenge for the non-biased qualitative measurement of the plastids. Nevertheless, it was considered worth mentioning that a clear qualitative difference was appreciated. Despite the above, there was a qualitative difference, with an apparent increase in the number of chloroplasts in the guard cells of the parent plant and mixoploid plants in comparison to the haploid lines (Figure 3). 

The length of the stomatal guard cells of the two haploid lines showed statistically significant differences from PHaploid#3 and the parent plant. In the case of stomatal width, all three putative haploid lines separated from the parent plant. All of the material used for stomatal morphology comparisons was taken from plantlets kept in vitro to eliminate possible bias related to environmental variability. 

Despite the time-consuming and specialized labor required for plant karyotyping, chromosome counting is still the most reliable and cost-effective method to confirm ploidy levels [21]. In this study, we found that the *V. planifolia* parent plant (AC110) showed variable somatic numbers of chromosomes, with 2n counts of 28 and 32 for different cells in the same tissue. Conversely, the gametic number of chromosomes from the pollen cells of the parent plant indicated a stable number of n = 16 chromosomes. We also used another *V. planifolia* accession, ”Daphna”, as a reference, and the chromosome count resulted in a constant number of 28 in all of the somatic cells and n = 14 chromosomes in all of the pollen cells (Table 1). Our results are in agreement with the previously reported chromosome studies for *V. planifolia* [18,19,22,23].

Endoreplication (or endoreduplication) is the process of nuclear genome duplication without the subsequent cell division, resulting in an overrepresentation of the genetic material in the cell [24]. In plants, endoreplication has been related to cell specialization, cell elongation, organ development, and pathogen or symbiotic interactions, as well as succulent tissue development [25]. Partial endoreduplication is the process in which only part of the genomic DNA is duplicated. So far, it seems that this differential duplication of genomic DNA is restricted to the orchid family and has been reported to occur in vanilla [19,24,25]. The meristematic region lacks endoreduplication cells, providing unique clean peaks when used for flow cytometry. We used gametic tissue (pollen cells) as a control for the chromosome counting. Nodal tissue was found to be challenging, and meristem excision was not achieved in dormant nodes. Actively growing root tips were found to be the most suitable alternative, as well as meristematic regions in actively growing tips.

Genome size estimation using flow cytometry has a higher throughput than chromosome counting. With appropriate standards, genome size can be used to indirectly determine ploidy levels for many crops. Nevertheless, this technique has been deemed challenging in vanilla plants because of partial endoreduplication, making the use of chromosome counting necessary. To obtain precise flow cytometry genome size estimations in vanilla orchids, actively growing tissue in the meristematic region must be used. This tissue will not have undergone partial endoreduplication and will render accurate estimations of genome size. Research has shown that leaves or even dormant buds produced broad fluorescence peaks or multiple peaks representing the different stages of partial endoreduplication [19,24]. 

In the case of haploid lines, multiple peaks can become confusing and ambiguous. We succeeded in the precise dissection of meristematic tissue and were able to reduce the endoreduplication peaks and focus on un-reduplicated cell counts. It is fundamental to avoid any mature succulent tissue, a good indication of which is tissue pigmentation; green tissue has usually undergone endoreplication, whilst white/transparent tissue in the meristematic region has not. The two confirmed haploid lines provided narrow peaks in comparison to the mixoploid line PHaploid#3, which provided several peaks of similar abundance with broad standard deviations amongst the replicated reads. Despite the challenges, flow cytometry presented as the most accessible approach to compare genome sizes, and as a great tool to assess haploid lines if the correct tissue is used.

Three putative haploid lines were recovered from the *V. planifolia* parent plant (AC110). Two of the lines (PHaploid#1 and PHaploid#2) were verified to be haploids, and the remaining line (PHaploid#3) was found to be a mixoploid resembling a diploid vanilla plant. Producing haploid plants is the first step towards developing double haploids that can be used in breeding strategies. Double-haploid lines serve as a great source of stable sexual seed production. Chromosome doubling of haploid plants has been achieved in many plant species using antimitotic agents such as colchicine. It is important to note that colchicine is also a mutagenic and results in other alterations to the genetic information of the plant [26]. Another chemical treatment known to result in chromosome doubling is the use or oryzalin (3,5-dinitroN4,N4-dipropylsulfanilamide), which has been regarded as an alternative to the multiple side effects of colchicine [27]. 

Successful attempts to induce polyploidy have been reported in many plant species [10,28,29,30,31]. On the other hand, it is also known that spontaneous chromosome doubling happens in plants. The mechanism governing this spontaneous doubling of the genome has not been thoroughly explained, but it has been speculated that it might happen by means of unreduced gametes, endomitosis, nuclear fusion, or endoreplication [29]. In orchids, the spontaneous genome doubling seems to be quite common, achieving rates of more than 30% of the regenerated tissue culture plants resulting in polyploids in Phalaenopsis [32]. Similar results have been reported for interspecific hybrids between Laelia and Cattleya parents [33]. The later report mentioned endoreduplication as a trait that increases the possibility of spontaneous polyploidization. Vanilla plants also present endoreduplication, opening the possibility of spontaneous genome doubling. 

## 4. Materials and Methods

### 4.1. Plant Material

**Figure 6:**
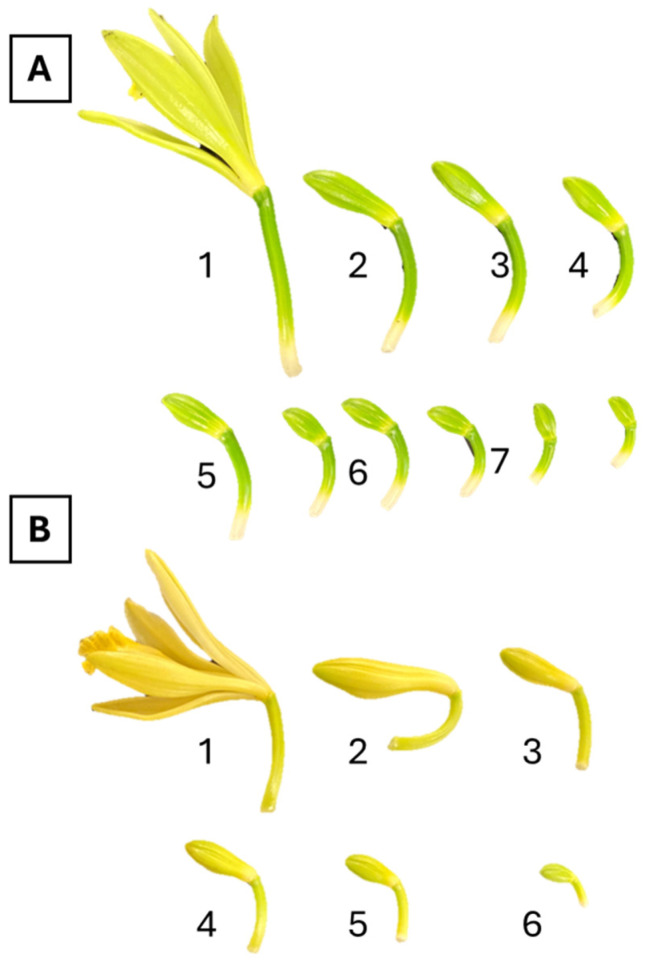
Floral stages used as sources of explants for haploid development. The number was assigned based on the size of the flower bud, with number one being the largest size (unpollinated open flower harvested on the day of opening). (**A**) Flower bud samples from *V. planifolia*. (**B**) Flower bud samples from *V. pompona*.

Two accessions of *Vanilla planifolia* (AC110 and AC67) and one accession of *Vanilla pompona* (AC72) from the University of Florida’s vanilla breeding program were used for haploid development. Floral buds at specific developmental stages were dissected, surface disinfected, and cultured in growing media (Figure 6). 

**Figure 7:**
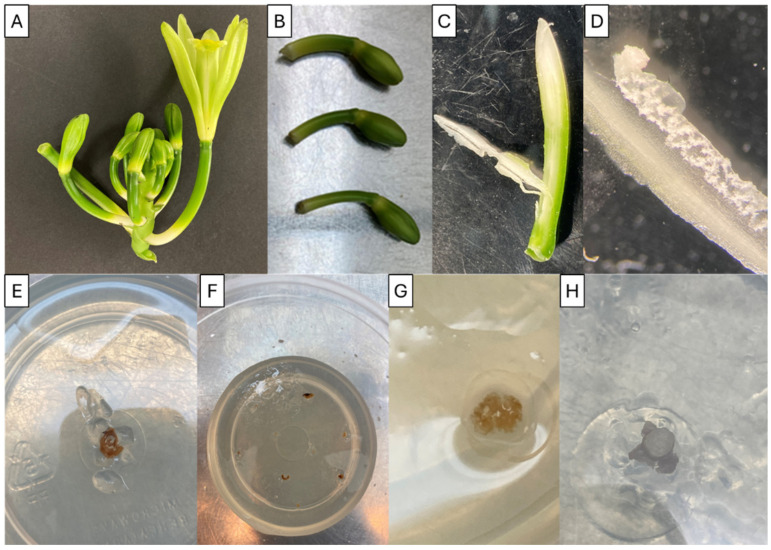
Overview of the initiation process for haploid development in vanilla: (**A**) *V. planifolia* floral raceme. (**B**) Closed floral buds. (**C**) Excised ovary with placental section. (**D**) Placental ovary section with ovules under stereoscope ×20. (**E**) Planted placental ovary section in growing medium. (**F**) Planted placental ovary sections in growing medium. (**G**) Developing ovaries in placental ovary section in growing medium. (**H**) Swollen unfertilized ovary excised and planted in growing medium.

Placental ovary sections were extracted from the superficially disinfected floral buds and cultured in O139 Orchid Maintenance/Replate Medium (PhytoTech Labs, https://phytotechlab.com/mwdownloads/download/link/id/314, accessed on 6 June 2024) supplemented with 1 mg/L of 6-benzylaminopurine, 0.1 mg/L of ABL02 Antibiotic–Antimycotic Solution (Caisson Labs (https://caissonlabs.com/product/antibiotic-antimycotic-solution-100x/, accessed on 6 June 2024), and 6 g/L of agar. Planted containers were placed in the dark at 24 °C and transferred to the same fresh media weekly until the formation of PLBs (~8 months since planting) (Figure 7). 

PLBs were transferred to fresh O139 medium supplemented with 10% liquid coconut endosperm (*v*/*v*) under a 12 h photoperiod at 24 °C, replanting monthly, which resulted in complete plantlet formation. Once plantlets developed, they were further propagated through micro-cuttings and kept in O139 media plus 10% liquid coconut endosperm to sustain a clonal population to be used for ploidy confirmation and further research. Chromosome counting, stomatal morphology analysis, and flow cytometry were employed for ploidy verification.

**Figure 8:**
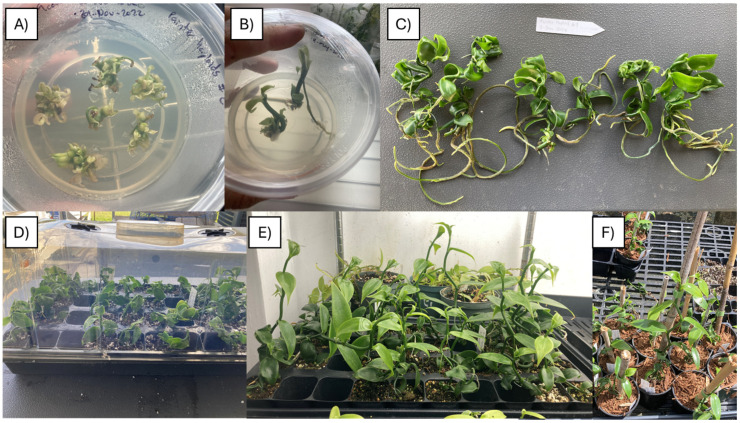
Ex vitro adaptation of putative haploid lines: (**A**) Shoot clusters under O139 media supplemented with BA. (**B**) Plantlets under O139 media supplemented with liquid coconut endosperm. (**C**) Fully developed plantlets ready for hardening. (**D**) A 36-cell tray with a plastic dome for ex vitro adaptation. (**E**) Plantlets without plastic dome. (**F**) Adapted putative haploid lines in the shade house.

**Figure 9:**
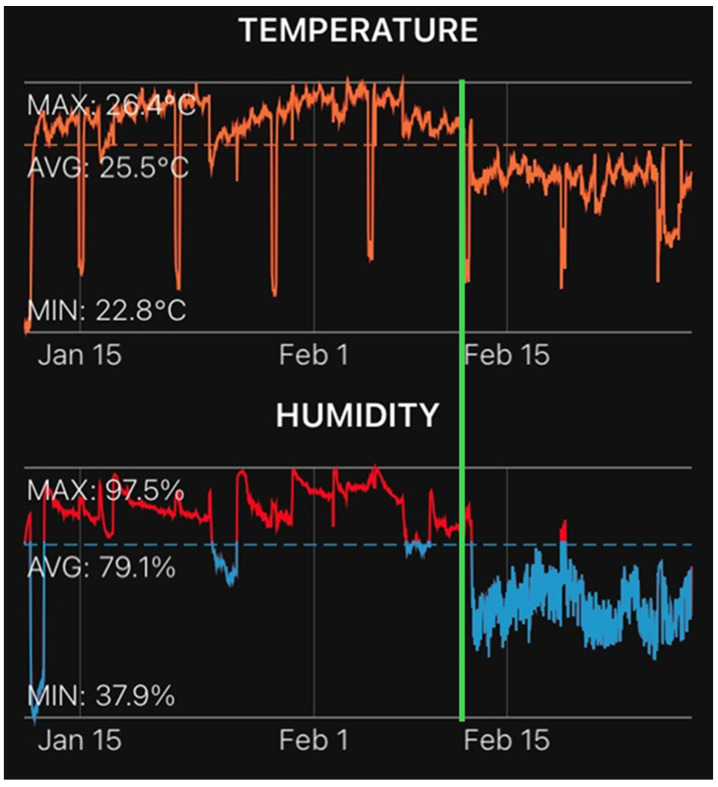
Temperature and humidity regimen during the hardening process of putative haploid lines. The green line indicates the time when the plastic dome was removed one month after planting.

Fully developed plantlets were removed from the containers. The growing media was washed from the roots, and the plantlets were placed on a 36-cell growing tray with a plastic dome inside a growing chamber (Figure 8). The relative humidity was kept close to 80%, and the temperature was maintained at an average of 25 °C during the hardening stage (Figure 9). One month after planting ex vitro, the plastic dome was removed, and relative humidity was allowed to fluctuate between 79% and 37%, while the temperature was also allowed to fluctuate between 25 °C and 22 °C (Figure 9).

### 4.2. Chromosome Counting

Chromosome counting was conducted on root tips using aceto-orcein staining. Actively growing root tips from in vitro plants of each putative haploid line, plus the parent plant, were harvested by excising 1 cm from the tips of healthy, actively growing roots. Excised roots tips were pre-treated in an 8-hydroxyquinoline solution at a concentration of 0.002 M for 2 h at room temperature (21 °C). Subsequently, the root tips were transferred to a hydrochloric acid solution at 1 N concentration at room temperature for 10 min. After acid hydrolyzation, the root tips were transferred to an aceto-orcein solution at a concentration of 2% *v*/*v* for one hour in the dark. Stained roots were dissected, and the stained tips were transferred to a microscope slide and squashed with a glass slide cover for chromosome spreading [34,35,36]. Micrographs of root cells with stained chromosomes were taken using an Axiovert S100 inverted microscope coupled with a Zeiss Axiocam 705 mono digital camera. Pollen cells were used as controls. Chromosome staining was executed following the aceto-carmine protocol described by Windham et al. [37]. 

### 4.3. Stomatal Morphology Comparison

Stomatal morphology was examined using microscopic techniques. Three leaves from in vitro plants of each putative haploid line plus the parental plant were harvested, and longitudinal cuticle sections of the abaxial side were mounted in a 0.1% *w*/*v* aqueous solution of AgNO_3_ on microscope slides [12]. Slides were observed under an Axiovert S100 inverted microscope at 2000× magnification, and fifteen stomata per line were micrographed using a Zeiss Axiocam 705 mono digital camera. Micrographs of the stomata were measured for length and width using ZEN 3.5 (blue edition) image analysis software. The variance in the stomatal size data was analyzed by ANOVA coupled with a post hoc Tukey’s honest significant difference test with an alpha value of 0.05. 

### 4.4. Flow Cytometry Genome Size Estimation

Flow cytometry analysis was performed using internal standards to estimate genome size and confirm ploidy levels. An Attune NxT acoustic focusing flow cytometer coupled with an autosampler was used to estimate the genome size of each sample using both “Jupiter” (*Oryza sativa* L.) (Reg. No. CV-119, PI 639742) rice, wheat landrace “TW0190” and Barley “Butta 12” (*Hordeum vulgare* spp. *vulgare*).

The genome size of each putative haploid line and parent plant (control) was measured in triplicate, and the mean value was reported. A portion of the growing tip consisting of the meristem and the two first leaf primordia was used as an explant for the vanilla samples to avoid endoreduplication. The tissue was dissected and placed in a plastic Petri dish with a similar amount of leaf tissue of the internal standard. In the case of the rice internal standard, the white base of the plant was also used to avoid endoreduplication peaks. Both sample and internal standard tissues were placed on clean plastic Petri dishes with two drops of General Purpose Buffer plus propidium iodide and RNAse A, before being chopped thoroughly with a new sharp razor until tissue homogenization [38]. 

## 5. Conclusions

This study presents a comprehensive methodology for the development and verification of haploid lines in *V. planifolia*. The identified haploid lines hold promise for accelerating breeding programs and improving vanilla cultivation. Further research is warranted to double the chromosome number to develop elite double-haploid lines for breeding and/or for stable hybrid seed production of vanilla for commercial use.

The successful development and verification of haploid lines in vanilla demonstrates the feasibility of this approach for breeding programs. The use of multiple techniques for ploidy verification ensures robust results, despite challenges such as partial endoreduplication. These haploid lines offer a valuable resource for future breeding efforts aimed at enhancing vanilla production and quality.

To the best of our knowledge, this is the first report of haploid plants’ development in vanilla, representing a novel addition to the breeding tools available for the advancement of vanilla orchid breeding. 

## Figures and Tables

**Table 1:** Survival rates during hardening of putative haploid lines.

Genotype	Start Hardening	End Hardening	Hardening Time (Months)	Initial Number of Plants	Final Number of Plants	Survival Rate (%)
PHaploid#1	3 January 24	28 February 24	1.8	6	6	100
PHaploid#2	3 January 24	28 February 24	1.8	12	10	83
PHaploid#3	3 January 24	28 February 24	1.8	10	10	100

**Table 2:** Chromosome counts for parent plant (AC110), putative haploid lines and extra *V. planifolia* reference accession (Daphna).

Sample	Number of Chromosomes	Ploidy
PHaploid#1 root tips	14	n
PHaploid#2 root tips	14	n
PHaploid#3 root tips	32	2n
AC110 root tips 1	28	2n
AC110 root tips 2	32	2n
AC110 pollen	16	n
Daphna root tip	28	2n
Daphna pollen	14	n

**Table 3:** Genome size estimation of the parent plant (AC110) and putative haploid lines.

Sample	Mean Estimated Genome Size	SD	Ploidy
AC110	4.468	0.127	2x
PHaploid#1	1.843	0.116	x
PHaploid#2	1.882	0.115	x
PHaploid#3	3.030	0.929	Mixoploid

## Data Availability

Data are contained within the article.
